# QEOSA: A Pedagogical Model That Harnesses Cultural Resources to Foster Creative Problem-Solving

**DOI:** 10.3389/fpsyg.2019.00833

**Published:** 2019-04-24

**Authors:** David Yun Dai, Huai Cheng, Panpan Yang

**Affiliations:** ^1^ University at Albany, Albany, NY, United States; ^2^ China Preschool Education Group (Beijing), Beijing, China

**Keywords:** problem-solving, indigenous epistemologies, developmental constraints, creative mind-set, pedagogical design

## Abstract

The nature of creative thinking is complex and multifaceted, often involving cognitive processes and dispositions modulated by implicit cultural belief systems and ways of thinking. In this article, we build on existing research on the relations of creative thinking and culture, and explore how specific cultural resources can be harnessed to foster creative problem-solving in education. We first review the recent changes in our understanding of creative thinking, from an exclusive focus on cognitive processes to a more inclusive view of creative problem-solving as socially negotiated and culturally modulated, carrying important cultural functions. We then introduce a pedagogical model, QEOSA, to illustrate how cultural resources, particularly culture-specific ways of thinking about the world, can be harnessed to foster creative thinking in education, and what developmental and pedagogical considerations are involved to make it effective. We finally conclude this article by indicating the value of this line of work that integrates psychological, cultural, developmental, and educational principles in fostering the development of a creative mind-set with relevant knowledge, skills, dispositions, and values.

## Fostering Creative Thinking: Cognitive Considerations

Creativity is often defined in terms of a psychological process leading to novel and useful ideas and products (Plucker et al., 2004). While distinct cognitive processes and mechanisms are identified (Finke et al., 1992), they are always associated with affective and conative forces that provide the necessary impetus, that is, energy, purpose, and direction for such endeavor (Dai and Sternberg, 2004). In addition, as long as this process involves multiple individuals interacting with one another in such an undertaking, the process is socially engendered and culturally facilitated (Sawyer, 2012). Thus, connecting cognition, creativity, and culture entails the recognition that the creative process is complex and multifaceted.

There has been a bulk of research on creative thinking and creative problem-solving (see Mumford and Gustafson, 1988; Plucker et al., 2004 for reviews). It is difficult to exhaust all possible processes and mechanisms that help define and solve problems in a creative way. There are two prominent issues, however, regarding creative thinking and problem-solving. One is how to overcome rigidity in thought and exercise cognitive flexibility, particularly when a problem is ill-defined; that is, the nature of the problem as well as the solution paths is not clear (Spiro et al., 1991). In real life, individuals can be easily the victims of mental sets and entrenched views that create tunnel vision and dogmatism (Ambrose et al., 2013). Besides self-serving biases and interests, there are important cognitive sources of rigidity in thinking. We tend to think about issues in terms of conflicts and contradictions, for example, pitting economic development against environmental protection, as if one is also gained at the cost of the other. Cast such thinking in game theory, all games are zero-sum games: if the other side wins, you lose. This is a cognitive trap easy to fall into, even for scientists and scholars; if a claim is true, then an alternative claim is always false (e.g., the perennial nature–nature debate in psychology; see Dai, 2012). In either case, breaking mental sets (Luchins and Luchins, 1970) is essential for finding out better alternative possibilities and solutions, rather than rigidly adhering to a fixed problem representation and solution path (Dai and Cheng, 2017). For example, to overcome conceptual entrapment, we can alternatively view environmental protection as a win-win opportunity for job creation and reindustrialization; or on the nature-nurture issue, we can see nurture sometimes transcends nature, and other times reveals nature, or view both as making up one inseparable functional and developmental system (e.g., Gottlieb, 1998).

In addition to initial problem framing, a subsequent issue is how to seek better solutions by carefully constructing a problem representation that identifies and satisfies relevant goal-related constraints in reaching a solution. Researchers have reached a consensus that creativity involves both divergent and convergent thinking (Treffinger, 1995; Cropley, 2006; Runco, 2010), and that creativity is not just free ideation or association but entailing knowledge of a problem and exercise of strategic thinking about tackling a problem (Mumford and McIntosh, 2017). For that matter, while holding multiple, sometimes competing, perspectives is important, a crucial step moving problem-solving forward is to critically analyze these options and negotiate a viable solution through gathering and integrating information.

In sum, envisioning multiple possibilities while holding a critical perspective is the key to creativity (Baron, 2000; Langer, 2012); we define this way of approaching specific real-world problems as a *creative mind-set*, a concept we will elaborate on in later sections. While novelty of a solution comes from cognitive flexibility that generates alternative perspectives and possibilities, usefulness and appropriateness come from careful evaluation of alternatives that help select more viable and optimal ones. Because creative problem-solving in the real world is often knowledge-rich rather than knowledge-lean (Finke et al., 1992; Ericsson et al., 2006), transfer of learning and problem-solving across situations through analogical mapping or rule-based reasoning becomes a relevant issue as to *how one learns to be creative*. As we shall demonstrate later with QEOSA, a pedagogical model, fostering creative problem-solving through education can be firmly based on such scientific understanding of underlying social-cognitive processes.

## Fostering Creative Thinking: Cultural Considerations

There are many ways of defining culture. For the purpose of this article, we view culture as conventions and norms that regulate thought and action among members of a particular group. In an important way, it is an “invisible hand” behind people’s attitudes, thoughts, mind-sets, and behaviors (Triandis, 1990). For example, norms in individualistic cultures encourage self-expression, and norms in collectivist cultures expect conformity and self-control. There is evidence that individuals in loose cultures are more likely to succeed in creative tasks than individuals in tight cultures (Chua et al., 2015), and tight cultures are less receptive to novel ideas deviating from accepted ways of life or thinking (Shane, 1992; Jones and Davis, 2000; Niu and Sternberg, 2001). Furthermore, individualistic cultures value originality, an essential component of creativity (Torrance, 1972), compared with collectivist cultures (Bechtoldt et al., 2010).

Arguments have been advanced, however, against such broad-brushed generalization. In a more nuanced manner, Arieli and Sagiv (2018) showed through a series of experiments that congruence between cultural mind-sets and problem types matters in effective problem-solving. Kharkhurin (2014) suggested that Western ideology highlighting individualism and Eastern ideology stressing the collective or common good may yield qualitatively different views of what constitute creative solutions (see also Niu and Sternberg, 2002). Shi (2008) challenged the view that traditional Chinese culture, a collectivist culture, hinders creativity. He identified essential characteristics, such unity of the person and the universe (天人合一), the value of harmony and “middle way” (贵中尚和), and moral reflection (知耻自省), among others, as important for a creative personality. His view of creativity has a distinct ethical and moral overtone, similar to Kharkhurin’s conception of creativity as involving utility values specific to particular cultures. In the same vein, Hennessey (2017) advocated a systems view of creativity with a distinct cultural dimension: creative problem-solving serves a cultural agenda of solving real-world problems of cultural importance. Viewed this way, an emphasis on the person and the universe as forming a unity (in Chinese culture) rather than an antagonistic relationship seems to be more productive in terms of being harmonious with nature for an agricultural economy. There are distinctive culture-specific epistemic beliefs or indigenous epistemologies (Dai, 2015) in terms of what constitute *reliable and viable ways or strategies to comprehend the realities and solve real-world problems*. These belief systems can either operate in an implicit manner, embedded or encrypted in a culture’s natural language and social practices (Masuda and Nisbett, 2006; Paletz and Peng, 2009), or be articulated by intellectual leaders as mottos or scripted teachings spread out throughout a culture.

Are these norms, conventions, and belief systems necessarily more or less creative in serving their respective cultures? We differ from some researchers who set out to determine whether specific epistemic beliefs in a culture (e.g., dialectic thinking) can or cannot facilitate creative thinking (e.g., Paletz et al., 2018). We believe that epistemic beliefs serve as heuristics rather than algorithms in problem-solving situations. To fashion our strategies for promoting creativity, we asked what part of Chinese culture helps people break their rigid, dichotomous (i.e., either-or thinking) mind-set when dealing with practical dilemmas or conceptual conflicts, and what kind of epistemic beliefs in Chinese culture facilitates cognitive flexibility and creative ideation. In other words, there are a set of norms, conventions, belief systems that can be made explicit and “harnessed” as cultural resources to foster creative thinking. As a result, we found rich connections between what the psychology literature we reviewed earlier helps us conclude, and what was conveyed from two most influential schools of thought in Chinese history: Confucianism and Taoism.

The Confucian golden mean (中庸), or the value of harmony and “middle way” mentioned earlier, is an ethical rule that helps balance competing social concerns and maximize gains for all concerned parties with competing priorities (i.e., for the common good). In contrast to the tendency in Western cultures for stressing and polarizing differences and conflicts, the principle of golden mean seeks harmony and unity. It is based on the conviction that, rather than destined to be a zero-sum game, optimal solutions can be found for complex social problems with competing and conflicting priorities and claims. In effect, it is similar in spirit to Sternberg’s (1999) balance theory of wisdom. The influence of the principle of harmony is even embodied in the ying-yang logo showing the seamless complementarity and perfect harmony of the apparent opposites. Indeed, the logo itself evidences originality and creativity. Pervasive cross-cultural differences in ways of thinking can be found in research. For instance, Peng and Nisbett (1999) compared college students from China and United States and found that American students tend to engage in adversarial dispute resulting in polarized viewpoints, whereas Chinese students are more likely to consider both sides of competing or contradictory arguments as valid to some extent and thus have a tendency to seek the “middle road” (see also Masuda et al., 2018; see Nisbett, 2003 for a general review).

In comparison to Confucians concerned mainly with ethics, Taoists (e.g., Lao Zhi and Zhuang Zhi) took a more epistemological approach; they tried to make people aware of language entrapment and entrenched conceptual schemas that prevent them from thinking freely and adaptively in an ever-changing world. They maintained that breaking language and conceptual barriers is the only way to achieve free thinking, hence creativity (see Feng, 1988). Taoism inherited the essence of the Book of Change in that it views the world as constantly changing, a kind of dynamism based on ying-yang dialectics that defies static description (see Dai, 2015). Again, this idea finds support from the contemporary psychology literature showing the impediments of entrenched perspectives or mind-sets on creative problem-solving (Frensch and Sternberg, 1989; Kameda and Sugimori, 1993; Ohlsson, 2011; Brockner and Rubin, 2012).

A crucial question from a practical point of view is that, if the Chinese conventions and norms in thought and action are implicitly functional in people’s everyday life, is it possible to articulate and harness them to foster creative thinking and problem-solving in education, especially in formative years of human development? After all, if creativity is truly “an important vehicle for cultures to advance their purpose” (Hennessey, 2017, p. 343), the process of enculturation should involve cultivation of such potential. This is precisely what we are trying to accomplish with QEOSA, to articulate and formalize an implicit aspect of culture for an educational intervention aiming to develop a creative mind-set capable of envisioning multiple possibilities while holding a critical perspective (Langer, 2012). Our work was inspired by Confucianism in the sense that creative solutions to complex real-world problems entail a balancing act for the common good. It was inspired by Taoism in the sense that, to achieve an optimal solution, one has to break loose the language and conceptual entrapment, particularly the either-or dichotomous mental set. In this way, the Chinese cultural ideas we introduce here, generated more than 2000 years ago, serve as *heuristics* or *norms* to guide thinking and problem-solving. To fully implement this agenda, there are developmental and pedagogical considerations, to which we now turn.

## Fostering Creative Thinking: Developmental and Pedagogical Considerations

Masuda et al. (2018) explored the developmental ramifications of enculturation in terms of cultural ways of thinking. It is clear that cross-cultural differences in cognitive processes mediated by cultural influences must have a developmental underpinning; that is, they have to do with the use of specific symbolic systems (e.g., language) as well as patterns of social interaction over time in formative years in shaping the way individuals feel and think, namely, enculturation (Peng and Nisbett, 1999). A question can be raised as to whether we can deliberately cultivate a *creative mind-set* in formative years, and what are psychological mechanisms that mediate developmental changes involved. Indeed, to postulate that young children can formulate creative solutions to complex and ill-defined problems almost violates the developmental canon that thinking and reasoning become more sophisticated only when one reaches adulthood (see Grossmann, 2018 for a review of Reigel’s and Perry’s theories). This is why it is more common to see well-defined problems featured in early childhood education; it was not until recently that the issue of designing a learning environment featuring ill-defined problems and projects was brought to public attention (e.g., Resnick, 2017).

Decades ago, Torrance (1972) analyzed and summarized 133 studies that were designed to examine whether children can be taught to think more creatively. His review indicated that it is possible to teach children to think more creatively. In addition, several case studies demonstrated that creativity can be cultivated in formative years. Although under the influence of developmental theory (e.g., Piaget, 1950/2001), people tend to see young children as incapable of hypothetical thinking, Craft et al. (2007) showed that creative learning of children aged 3–7 can be enhanced by what she called *possibility thinking*, a process by which children are prompted to switch modes of thinking from “what is” to “what might be” (Craft, 2002). Although analogical transfer of problem-solving is considered difficult even for college students, Brown and her colleagues (e.g., Crisafi and Brown, 1986) showed when structured properly (e.g., ensuring that children noticed structural similarities between two problems), children as young as 2–3 years old are capable of applying a reasoning rule and making analogical transfer in problem situations, which is a basic mechanism for far transfer and creative problem-solving (Mayer, 1992; Lohman, 1993). Ansburg and Dominowski (2000) found that children having creativity training solved 14–24% more problems than a control group. Together, they suggest that creative thinking in formative years can be enhanced by carefully designed activities.

Developmental considerations go beyond just age-appropriateness of specific knowledge, skills, and dispositions we expect children to master in order to exercise creative thinking; they also draw our attention to the nature of creativity from a developmental point of view. Many scholars (e.g., Mumford and Gustafson, 1988; Sternberg and Lubart, 1991; Amabile, 2001; Perkins and Tishman, 2001) argue that intelligence or creativity is not a unitary ability or skill to be developed, but reflects a combination of knowledge, skills, values, and personal dispositions, coupled with an environment conducive to acts of intelligence and creativity. Viewed this way, enculturation of a *creative mind-set* capable of envisioning multiple possibilities while holding a critical or evaluative stance (Langer, 2012) is a long-term developmental proposition that involves the growth of the whole mind, not merely skill sets. According to Vygotsky’s (1978) social-cultural theory, such individual development involves an internalization process involving prolonged co-construction of cognitive apparatus with more competent peers or adults (see Moran and John-Steiner, 2003). An important pedagogical implication is that, although a “skill training” approach to enhancing creativity can be useful, in formative years of individual development, a growth-oriented approach to nurturing a creative mind-set through *enculturation* is more viable and effective.

Consistent with the view enhancing creativity through enculturation, Torrance (1972) argued that the most successful method of teaching children to think creatively is to consider their cognitive and emotional functioning, give them sufficient structure and motivation, and provide opportunities to engage, practice, and interact with teachers and other children. These instructional strategies form the core of a pedagogy for fostering children’s creative thinking. For example, at the beginning, teachers need to evoke children’s knowledge and emotional experiences with a presenting problem, and then motivate children to think creatively and scaffold the creative ideation and action through social interaction such as brainstorming. More recently, metacognitive monitoring of creative work is also emphasized as part of this pedagogy (e.g., Scardamalia and Bereiter, 2006). All these features are incorporated into QEOSA.

**Figure 1:**
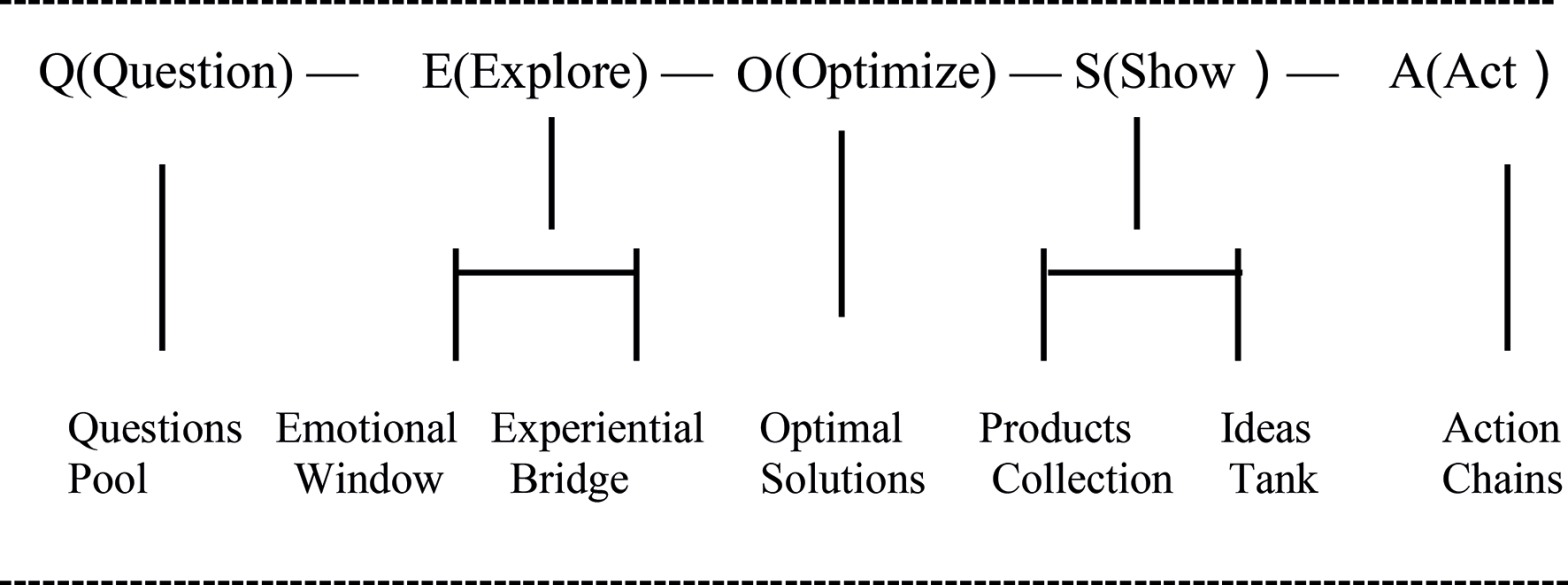
QEOSA: A pedagogy model specifying five phases and seven processes.

More specific to learning to be creative problem solvers is the issue of transfer: how to develop a unique set of knowledge, skills, dispositions, and values, a mind-set that is responsive to a range of problem situations calling upon creative solutions, and capable of generating novel and useful solutions. This was the impetus that drove the development of a pedagogical model, QEOSA (Figure 1; Cheng, 2012).

## Qeosa as a Pedagogical Model of Fostering Creativity in Formative Years

QEOSA, standing for Question, Explore, Optimize, Show, and Act, is a pedagogical model developed in the context of preschool education, aiming to nurture creativity in formative years with children of age 3–6 years (Cheng, 2012). Historically, QEOSA was developed as an alternative to teaching divergent thinking. Divergent thinking focuses on ideational fluency and functional flexibility, elaboration, and originality whereas QEOSA emphasizes finding optimal solutions to real-life dilemmas. A focus on optimal problem-solving represents a distinct quality-over-quantity strategy compared to the divergent thinking paradigm of creativity, which originated with Guilford’s (1950) conception of creativity, and the evolution model of creativity: blind variations followed by selective retention (Campbell, 1960; see also Simonton, 2003).

In keeping with the current practice of using authentic occasions for fostering and assessing creativity (Treffinger, 1995; Mumford and McIntosh, 2017), QEOSA uses practical problems accessible to preschool children as the main tool for enhancing and assessing creativity rather than more contrived traditional divergent thinking tasks.

While inspired by the Chinese cultural tradition embodied in Confucianism and Taoism in terms of seeking win-win solutions to social and practical problems and conflicts, and overcoming the conceptual entrapment of either-or dichotomous thinking, QEOSA integrates cognitive, developmental, and pedagogical considerations in informing a viable creativity pedagogy for young children. This way, cultural resources capitalized in QEOSA include but are not limited to culturally inspired ethical rule or epistemic stance; they include other norms (e.g., agency, collaboration, and responsibility) and conventions (e.g., children-initiated questioning, peer critiquing of new ideas) for promoting creative thinking. First, QEOSA structures learning experiences into five phases, with each phase having a distinct set of goals and activities; together, they constitute a steady progression toward a creative solution to some real-world problems. Second, QEOSA is a group-based pedagogy; peer interaction and collaboration as well as teacher-learner (in classroom) and parent–child interaction (at home) are the norm for scaffolding and synergistic play. Questions Pool, Products Collection, and Ideas Tank are built as public records of problem-solving for later productive use in a collective manner. Organized this way, QEOSA stresses the social (vs. solo) and co-constructed nature of creative problem-solving, which serves the important function of scaffolding optimal problem-solving. Third, to make all steps of problem-solving accessible and visible to young children, all learning activities involve pictures, tangible tools, and manipulatives. In the following section, we delineate each of the five phases of QEOSA in detail, illustrated by a case “Grandpa’s Misgivings” used in instruction.

### The Questioning Phase

The first phase of QEOSA is to generate a pool of questions raised by young children. For example, the case we introduce here, Grandpa’s Misgivings, was initiated by a question asked by one girl, Diandian: “My grandpa always forgot to take medication. What should I do?” This question brought forward a dilemma: her grandpa was troubled with forgetting (he is taking multiple medications with different schedules) but did not want to rely on her parents who had to constantly remind him of taking pills on time. Other children bring their own problems, questions, and observations to share. The teacher organizes a variety of opinions voiced by children and helps them clarify the problems in questions. Then the Questions Pool is created for later use.

### The Exploration Phase

**Figure 2:**
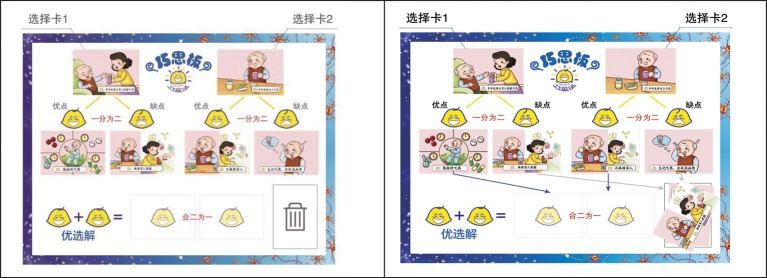
Q-Pad for the case of “Grandpa’s Misgivings”: The first figure shows constraints for optimal solutions, and the second figure shows an achieved optimal solution.

In this phase, children are asked to *brainstorm* ideas about the nature of a problem at hand as well as possible solutions. The exploration of various life dilemmas helps build the Experiential Bridge in terms of foregrounding children’s experience and factual knowledge (e.g., in the case of Grandpa’s Misgivings: illness, treatment, medicine, troubles with taking it on time, the help is not without costs). The next step is to guide children to think about advantages and disadvantages of each solution (e.g., Grandpa is prompted to take medication timely and correctly but the lives of mom and dad are disturbed, and *vice versa*). Two cards are presented to children when children brainstorm ideas of advantages and disadvantages of a solution. If a child identifies advantages of a solution, a happy face card will be given to her or him. Conversely, if a child identifies disadvantages of a solution, a sad face card will be presented. Such visible feedback evokes children’s emotions (i.e., opening *Emotional Windows)* and motivates cause-effect analytic thinking. The third step is to scaffold children’s ability to synthesize information for finding optimal solutions by providing a cost–benefit analytic scheme. As shown on Q-Pad presented in Figure 2, two solutions show both advantages and disadvantages, and children are encouraged to brainstorm ideas of creating a win-win solution, with advantages retained and disadvantages avoided (e.g., Grandpa will be able to take medication in time as well as correctly, while Diandian’s parents are free of the labor of constantly reminding her grandpa). The session ends with the teacher’s suggestion to children that they should go back home thinking and discussing with their moms and dads about a win-win solution. The sad and happy cards and Q-Pad serve two important pedagogical functions: to make cost–benefit analysis and paths to optimal solutions accessible to young children, to make children’s own thinking and reasoning visible to themselves. Related to the issue of design, Q-Pad provides a structure for navigating an ill-defined problem space with identifiable constraints (Welter et al., 2017).

**Figure 3:**
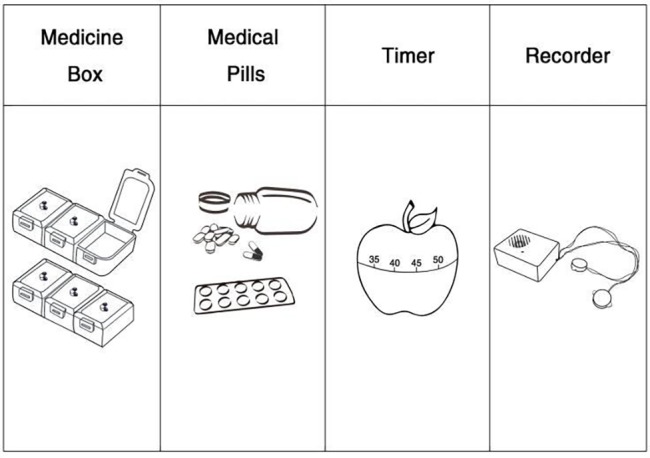
Toy tools used in the case of Grandpa’s Misgivings.

Introducing Q-Pad in early phases of problem-solving distinguishes QEOSA from the traditional approach: QEOSA imposes a structure (i.e., constraints for optimal win-win solutions) before brainstorming. The purpose of imposing structure early is to scaffold an evaluative (or critical) stance early toward solutions better than those involving a trade-off, a distinct *quality-over-quantity* strategy we mentioned earlier. This approach is developmentally appropriate given that young children are still fragile in terms of formulating logical thoughts (Piaget, 1950/2001). Research shows that identifying and satisfying goal-related constraints is central to problem-solving. Creative solutions sometimes rely on releasing assumptions of constraints (Ohlsson, 2011), and other times rely on identifying or setting up new constraints (Stokes, 2001). Optimal problem-solving that QEOSA promotes with dilemma cases involves satisfying multiple constraints so as to create a win-win condition. Medeiros et al. (2014) showed that imposing constraints, when done with the right doses, can improve creative problem-solving.

### The Optimization Phase

In this phase, the teacher first reminds children that they are striving for an optimal solution (i.e., seeking a win-win solution). The brainstorming session in the previous phase is followed by a critique session in which children challenge one another about validity and feasibility of the suggested solutions (i.e., whether they truly satisfy the cost–benefit constraints). Then, under the guidance of the teacher, different practical solutions are tried and tested, and children are guided to find tools and resources relevant to specific ideas. For example, in the case of Grandpa’s Misgivings, medicine boxes, (fake) medical pills, timer, and recorders become part of an optimal solution (see Figure 3). During this optimization process, children have to learn the design rule of combining these tools for a multifunctional design, another aspect of constraint satisfaction. Much “just-in-time” learning and the teacher’s scaffolding (but without telling) take place during this phase.

### The Show Phase

In this phase, children have to demonstrate, present, and share their ideas of how a dilemma can be solved. Although young children still do not have the skills to engineer their design ideas, they can show their solution by drawing or metaphorically using a seven-piece puzzle (a set of manipulatives for model building). The purpose of showcase children’s solutions is to develop children a sense of audience, ownership, and agency, both collectively and individually. Renzulli (2005) pointed out the motivational importance of addressing an audience and making a social impact with creative work, this point is often neglected by creativity researchers. Since the use of QEOSA in young children in China, more than a dozen plays based on real incidences of QEOSA-guided problem-solving have been scripted and performed by participating children on stage in front of classmates and parents as audiences. In the Show phase, some projects were enacted as a theatrical play; so far, a repertoire of 17 plays have been created. And others as the Collection of Products or Ideas Bank are showcased in children’s learning fair. The Show phase not only presents occasions that enhance children’s sense of agency and accomplishments; it is also a metacognitive moment to show how they managed to get this far.

### The Action Phase

In this final phase, the main task is to truly materialize the solution, including searching the market value of a solution. Parents and teachers help children search relevant information as to, for instance, whether the multifunctional medicine box in the case of Grandpa’s Misgivings has been produced before. If not, they would help children produce a real multifunctional medicine box as they conceptualized. In this case, children were eventually able to invent a multifunctional medicine box to materialize the optimal solution, capable of automatically prompting Grandpa to take specific medications. These activities form an Action Chain to help improve children’s practical skills, but more importantly, enhance a sense of entrepreneurship and achievement. Indeed, so far more than four dozen QEOSA-guided and children-made designs and products have been officially patented in China.

## How Qeosa Contributes to the Discourse Regarding Bridging Culture, Cognition, and Creativity

In the previous section, we presented in a nutshell how QEOSA as a pedagogical model was conceptualized and implemented in education for young children. Particularly important to this special issue is the question of how does it contribute to the current discussion of the role of culture in creative thinking and problem-solving? There are at least three ways in which QEOSA makes culture more prominent in nurturing creativity, with its emphasis on (1) a distinct set of norms and conventions for thought and action embedded in QEOSA, (2) the social and co-constructive nature of developing creative thinking, and (3) the enculturation of a creative mind-set instead of the training of a skill set.

### Culture as a Set of Norms and Conventions for Thought and Action Embedded in Pedagogy

While the sequence of Questioning, Exploring, Optimizing, Showing, and Acting resembles current models of creative problem-solving (e.g., Treffinger, 1995; Mumford and McIntosh, 2017), optimal problem-solving engendered by QEOSA carries a distinct set of norms (implicit or explicit) and conventions (i.e., built-in procedures) aiming to shape a creative way of thinking about social and practical problems, dilemmas, and conflicts. Viewed this way, what we consider as influences of Confucianism and Taoism is just part of norm-setting (e.g., the relentless search for a win-win solution, or avoiding either-or conceptual entrapment). More broadly, other values are embedded in QEOSA, such as taking initiative and exercising personal agency, collaboration, and responsibility (be ready to defend one’s hypotheses or proposed solutions). In addition, QEOSA also institutes a set of built-in routines (i.e., conventions), such as creating questions and ideas banks for collective use, building *Emotional Windows*, and facilitating *Experiential Bridges* to activate personal experiences and generate new understandings, as well as ensure sustainability of optimal problem-solving. Together, these norms and conventions help create a culture of problem-solving among preschool children, analogous to creating a community of learners and inquirers (Brown, 1994), for knowledge building and creative knowledge work (Scardamalia and Bereiter, 2006).

Historically, creativity has been viewed as having a fixed set of underlying cognitive processes regardless of cultural experience. This view is challenged (Hennessey, 2017). A pedagogy capable of enhancing creative thinking is one that is capable of preparing and positioning a mind to solve real-world problems. From this perspective, even a Western conception of divergent thinking as quintessential for creativity, starting with Guilford (1950), reflects a cultural norm characteristic of an individualist culture.

### Social and Co-constructive Nature of Developing Creative Thinking

A developmental corollary of the above argument is that the process of developing creative problem-solving has to involve enculturation that is social in nature; thinking and reasoning, such as maintaining a critical stance, finding multiple possibilities, and seeking win-win solutions, must be co-constructed and scaffolded in formative years. The exercise of optimal problem-solving under QEOSA helps children not only solve complex problems creatively, but also come to appreciate the complexities of the real world and untapped possibilities for the common good. Indeed, the social and intellectual aspects of individual development are intricately connected. As shown in our introduction to QEOSA, almost in every step of the way, children are constantly working with the help of more competent peers and adults. In exploration as well as optimization phases, ideas are generated and improved in such a manner that no individual alone can claim full credit for the outcomes. This approach is in sharp contrast to trait or cognitivist conceptions of the genesis of creativity.

### Enculturation of a Creative Mind-Set Instead of the Training of a Skill Set

Mind-set, a term popularized by Dweck (2006), implies a specific way of thinking about important aspects of the world and self. If the norms and conventions involved in a culture of problem-solving embedded in QEOSA provide a structure in support of the development of a creative mind-set (seeking multiple possibilities while holding a critical stance; Langer, 2012), the co-constructing and scaffolding of problem representation and optimal solution finding provides a social-cultural meditational process that helps shape such a creative mind-set over time, the way children learn to relate to and think about the world and themselves in a way conducive to creative contributions.

Such a mind-set surely includes cognitive skills, but it goes beyond divergent and convergent thinking to encompass world knowledge, epistemic stance regarding how the world operates, and how we should approach real-world problems and issues (e.g., how to avoid conceptual entrapment). It should also involve a sense of personal agency for making a difference, whether it involves significant others (e.g., in the case of Grandpa’s Misgivings) or the human race (e.g., the issue of global warming). A corollary of the argument for developing a mind-set rather than merely cognitive skills deemed essential for creativity is that an exclusive cognitive approach to creativity, devoid of experiential, affective, knowledge, and social bases, is untenable (see Dai and Sternberg, 2004, for a critique of cognitivism). From a developmental and pedagogical point of view, this point becomes even more important in formative years (i.e., childhood).

## Conclusion

Historically, creativity research has made many turns in focus, from person, to process, to product, and more recently, to place (or press; see Dai, 2013). Lubart (2017) even coined seven Cs in mapping out all the components involved. This article represents our attempt to make a case for the importance of culture in creative thinking and problem-solving, with a focus on how various lines of research, psychometric, cognitive, developmental, and psychosocial, can be integrated in fashioning a pedagogy of creative problem-solving and the enculturation of a creative mind-set. QEOSA is just a small step in this direction. The enculturation hypothesis advanced in this article is predicated on the notion of transferability of such a creative mind-set. Empirical effort has been made along this line. Much research is warranted to advance this line of inquiry for the sake of individuals as well as the vitality of society.

## Author Contributions

DD conceptualized the paper. HC provided pedagogical materials and research data. PY assisted in reviewing literature and writing.

### Conflict of Interest Statement

The authors declare that the research was conducted in the absence of any commercial or financial relationships that could be construed as a potential conflict of interest.
